# The Disrupted Mitochondrial Quality Control Network: A Unifying Mechanism and Therapeutic Target for Chemotherapy-Induced Multi-Organ Toxicity

**DOI:** 10.3390/biology15030230

**Published:** 2026-01-26

**Authors:** Yaling Li, Ningning Ding, Xiufan Liu, Qi Si, Yong Wang, Changtian Li, Yongqi Liu

**Affiliations:** 1 Gansu University Key Laboratory for Molecular Medicine & Chinese Medicine Prevention and Treatment of Major Diseases, Gansu University of Chinese Medicine, Lanzhou 730000, China; liyaling@gszy.edu.cn (Y.L.); 18640644108@163.com (Q.S.); 2 Key Laboratory of Dun Huang Medical and Transformation, Ministry of Education of the People’s Republic of China, Gansu University of Chinese Medicine, Lanzhou 730000, China; 3 First School of Clinical Medicine, Gansu University of Chinese Medicine, Lanzhou 730000, China; dnljc2304@163.com (N.D.); x112679@163.com (X.L.); 4 Basic Medical School, Gansu University of Chinese Medicine, Lanzhou 730000, China; wangyong@gszy.edu.cn

**Keywords:** mitochondrial quality control system, chemotherapy toxicity, active compounds, organ protection, multi-target therapy

## Abstract

Chemotherapy remains a cornerstone of systemic cancer treatment, yet dose-limiting toxicities—cardiotoxicity, neurotoxicity, and nephrotoxicity—affect 40–80% of patients, interrupt 20–30% of treatment cycles, and double long-term mortality. We propose that these seemingly distinct organ toxicities converge on a single mechanism: selective disruption of the MQC network. MQC comprises five interdependent modules—biogenesis, dynamics, mitophagy, proteostasis, and the recently characterized migrasome-mediated mitocytosis—collectively maintaining ATP supply, redox balance, and Ca^2+^ homeostasis in high-demand tissues. Chemotherapeutics such as anthracyclines, platinum agents, and taxanes simultaneously repress PGC-1α-driven biogenesis, hyperactivate Drp1-mediated fission, impair autophagosome–lysosome fusion, and inhibit mitocytosis, triggering mitochondrial collapse, ROS overflow, and cell death. This first-in-field review delineates organ-specific MQC pathways and catalogs druggable interventions—including small molecules, natural products, and nano-delivery systems—that restore MQC checkpoints. We present an integrated “MQC disruption–multi-organ toxicity–targeted intervention” framework, identifying Drp1 hyperactivation, late-stage mitophagy arrest, and mitocytosis inhibition as core therapeutic nodes. Targeting these pathways offers a promising strategy to decouple anticancer efficacy from off-target toxicity, potentially enabling optimized dosing, reducing treatment discontinuation, and improving long-term prognosis. Most MQC-targeted agents, however, remain in preclinical or early-phase trials.

## Simple Summary

Chemotherapy is essential for treating cancer, but it often severely damages vital organs such as the heart, nerves, and kidneys, leading to treatment interruptions and reduced quality of life. This study aimed to identify a shared cause of these toxicities and potential protective strategies. We found that the common driver is damage to the mitochondrial quality control (MQC) network—a system that maintains the health of mitochondria, the cell’s energy powerhouses. Chemotherapy disrupts five core components of this network. We also identified treatments, including certain small molecules, natural products, and nano-delivery systems, that can repair the MQC network and protect organs without compromising chemotherapy’s anticancer efficacy. These findings could help refine chemotherapy regimens, reduce side effects, and improve long-term outcomes for cancer patients, offering meaningful progress in clinical care.

## 1. Introduction

Chemotherapy remains a cornerstone of systemic cancer treatment, significantly improving survival across diverse malignancies. However, its clinical utility is often constrained by dose-limiting, multi-organ toxicities. Cardiotoxicity, neurotoxicity, and nephrotoxicity affect 40–80% of patients, with incidence varying by drug class and tumor type [1]. These adverse effects lead to premature treatment discontinuation in approximately 30% of cases and cause substantial long-term morbidity, severely impairing survivors’ quality of life [2]. For instance, late mortality risk is elevated 2–3-fold among long-term childhood cancer survivors, largely due to delayed toxicities [3].

Despite this significant clinical burden, management strategies remain inadequate. Dexrazoxane is currently the only FDA-approved cardioprotectant specific to anthracycline-induced cardiotoxicity [4], while no targeted therapies exist for conditions such as platinum-induced peripheral neuropathy [5]. This pronounced therapeutic gap underscores an urgent need to identify unified pathogenic mechanisms and develop broadly applicable organ-protective strategies.

Previous research has linked chemotherapy-induced injury to various pathways, including DNA damage, oxidative stress, calcium dysregulation, and inflammation [6]. Yet these mechanisms often appear tissue-specific and fragmented, failing to adequately explain the convergent toxicity patterns observed across metabolically active organs [7]. Growing evidence now points to the MQC network as a central, unifying target compromised by diverse chemotherapeutic agents.

The MQC system is an endogenous regulatory network essential for maintaining mitochondrial homeostasis. It comprises five functionally interconnected modules: mitochondrial biogenesis (MB), dynamics (fusion/fission balance), mitophagy (selective clearance of damaged mitochondria), mitochondrial proteostasis (protein folding and degradation), and the recently characterized migrasome-mediated mitocytosis—an autophagy-independent extrusion pathway via TSPAN4 for moderately damaged mitochondria [8]. The integrity of this network is particularly critical in high-energy-demand tissues, and its disruption emerges as a common initiating event in multi-organ chemotherapy toxicity, thereby positioning MQC as a pivotal target for cytoprotective intervention [9,10].

Unlike prior studies that predominantly focused on the effects of specific chemotherapeutic agents on individual modules of the MQC system, this review provides the first systematic integration of all five core MQC modules—with particular emphasis on the recently identified migrasome-mediated mitocytosis pathway [8]—to establish a unified “MQC disruption” framework for chemotherapy-induced organ injury. Previous research has largely overlooked the systematic elucidation of organ-specific molecular networks underlying MQC impairment, as well as the critical evaluation of how MQC-targeted interventions can balance antitumor efficacy with toxicity mitigation. These gaps have hindered the translation of mechanistic insights into clinically applicable strategies.

We propose that analyzing the convergent and tissue-specific mechanisms of MQC disruption—while addressing the balance between anticancer activity and organ protection—will establish a theoretical foundation for developing MQC-targeted strategies. This review elucidates how chemotherapy drugs induce multi-organ toxicity by simultaneously impairing all five MQC modules, clarifies organ-specific pathways such as cardiac TOP2β–PGC-1α and neuronal Drp1–COX2, and comprehensively summarizes MQC-targeted interventions including small molecules, natural products, and nano-delivery systems. It also critically examines how these strategies may decouple organ protection from antitumor efficacy, thereby widening chemotherapy’s therapeutic window. Ultimately, we aim to provide a unified theoretical basis for understanding chemotherapy-induced multi-organ injury, identify tractable MQC nodes, and inform the development of precision organ-protective strategies that balance efficacy with safety.

## 2. Core Mechanisms of the MQC System

Mitochondria are double-membrane organelles approximately 0.5–1.0 µm in diameter, playing central roles in energy metabolism and functional maintenance in eukaryotic cells [11]. They participate not only in critical biological processes, such as energy conversion, redox regulation, calcium homeostasis, cell cycle regulation, and programmed cell death, but their functional integrity is also susceptible to threats from various internal and external factors, including oxidative stress and DNA mutations [12]. Consequently, mitochondria have evolved a multi-level MQC system to dynamically regulate their morphology, number, and distribution, thereby adapting to environmental changes [13]. This MQC system integrates mechanisms such as membrane potential regulation, redox homeostasis, dynamic fusion and fission, and autophagic clearance to cooperatively preserve mitochondrial functional integrity, which is crucial for maintaining cellular homeostasis [14]. This system integrates the five core mechanisms of mitochondrial biogenesis, mitochondrial dynamics, protein homeostasis, mitophagy and migrasome-mediated mitocytosis, forming a cascade reaction of “biosynthesis (generation of healthy mitochondria)–dynamic segregation (isolation of damaged mitochondria)—protein homeostasis (repair of functional deficiencies)—autophagy/cytosolic excretion (clearance of abnormal mitochondria)”.

### 2.1. Mitochondrial Biogenesis

**Figure 1:**
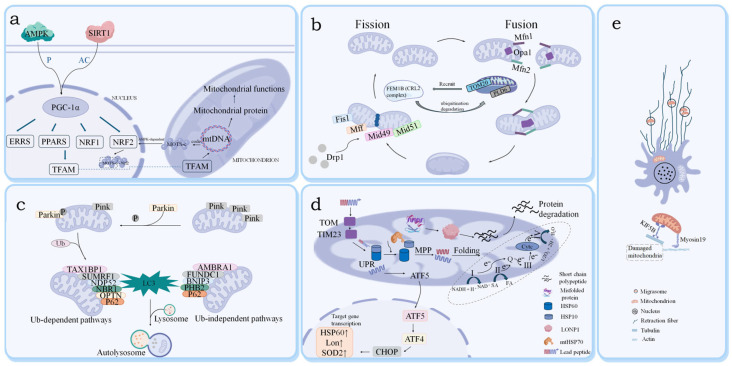
Schematic representation of the MQC system. (**a**) MB: Nuclear-localized PGC-1α is synergistically activated by AMPK (via phosphorylation) and SIRT1 (via deacetylation), which in turn induces the expression of transcription factors including ERRS, PPARs, and NRF1/2. These transcription factors promote the synthesis of mitochondrial TFAM; upon translocation into mitochondria, TFAM regulates the replication and transcription of mtDNA, ultimately sustaining the homeostasis of mitochondrial protein synthesis and mitochondrial function. (**b**) Mitochondrial dynamics: Mitochondrial fission is primarily mediated by Drp1. Drp1 is recruited to mitochondrial fission sites via outer membrane anchoring proteins (e.g., Mff, Fis1, MiD49/51), where it oligomerizes into helical structures to constrict and ultimately sever the mitochondrial membrane. This process is additionally modulated by ubiquitin ligase complexes such as CRL2^(FEM1B), which fine-tune mitochondrial dynamic homeostasis by degrading regulatory factors including TOM20. Mitochondrial fusion is mediated by Mfn1/2, which governs outer mitochondrial membrane fusion, and Opa1, responsible for inner mitochondrial membrane fusion. Notably, the degradation of RecQ1 also contributes to the regulatory modulation of this process. (**c**) Mitophagy: Ub-dependent pathways: Ubiquitination mediated by the Pink1–Parkin axis, in conjunction with molecules including TAX1BP1 and SUMO1, recruits LC3 and P62 to form autophagosomes. Ub-independent pathways: Molecules such as AMBRA1 and FUNDC1 directly recruit LC3 to initiate autophagy. Ultimately, autophagosomes fuse with lysosomes, leading to the degradation of damaged mitochondria. (**d**) Mitochondrial proteostasis: Mitochondrial proteostasis is regulated by full-process control of protein translocation (TOM/TIM23), folding (HSP60/10, LONP1), and degradation. UPRmt (ATF5, ATF4, CHOP) and antioxidant SOD2 jointly sustain mitochondrial protein function and redox homeostasis. (**e**) This schematic illustrates mitocytosis, a migrasome-mediated mitochondrial quality control process that couples mitochondrial homeostasis with cell migration: in a migratory cell containing a dotted nucleus, the mitochondrial network is polarized such that functional mitochondria reside in the central region, while damaged mitochondria are selectively targeted for removal; these damaged mitochondria are actively transported along tubulin-based microtubules toward the plasma membrane at the cell rear by the motor protein KIF5B, anchored to the actin cytoskeleton at the cell cortex via the adaptor protein Myosin19, and then undergo Drp1-mediated fission to produce fragments that are packaged into migrasomes at the tips of retraction fibers and ultimately expelled from the cell, acting as a conserved mechanism that allows diverse cell types to eliminate damaged mitochondria in response to various stressors and maintain cellular homeostasis.

MB is the core mechanism by which cells achieve mitochondrial self-renewal and functional remodeling through dynamic interactions between mitochondrial DNA (mtDNA) and nuclear DNA (nDNA) [15]. MtDNA encodes the 13 subunits of the electron transport chain (ETC) and some RNAs, while nDNA encodes and directs the transport of over 99% of mitochondrial proteins [10]. Its central regulatory factor, PGC–1α, is dynamically modulated by cellular energy status: under energy stress, elevated AMP/ATP levels activate AMPK phosphorylation, modifying PGC–1α; concurrently, elevated NAD^+^/NADH levels trigger SIRT1-mediated deacetylation. These processes synergistically enhance the cAMP–PKA–CREB signaling pathway [16]. Activated PGC–1α translocates to the nucleus, promoting the nuclear respiratory factor (NR–1/2) and mitochondrial transcription factor (TFAM, TFB1M/TFB2M) networks, thus facilitating mtDNA replication and nuclear-encoded protein synthesis [17]. Critically, this regulatory network establishes a positive feedback loop through estrogen-related receptor alpha (ERRα) to further enhance MB [10] (Figure 1a). This system integrates energy metabolism (AMPK/SIRT1), epigenetic modifications, and transcriptional regulatory networks to dynamically maintain mitochondrial quantity, function, and metabolic homeostasis [18]. Notably, MB-generated neo-mitochondria need to be integrated into the existing network through mitochondrial fusion, and their functional integrity is dependent on the proteostatic system being clear of aberrant proteins. The dynamic balance between MB and clearance pathways is critical for maintaining mitochondrial population stability.

### 2.2. Mitochondrial Dynamics

Mitochondria maintain a dynamic network through ongoing cycles of fission and fusion, which are essential for their morphology, intracellular distribution, and overall function [19]. In mammals, mitochondrial fusion occurs in two sequential steps mediated by specific GTPases. The initial stage of outer membrane fusion is facilitated by the mitofusins MFN1 and MFN2, which connect adjacent mitochondria; importantly, MFN2 also forms contacts with the endoplasmic reticulum (ER) [20]. Subsequently, the fusion of the inner membrane and the remodeling of cristae are driven by OPA1. The activity of OPA1 requires the proteolytic conversion of L-OPA1, which is membrane-anchored, into soluble S-OPA1, a process crucial for its fusogenic capabilities and, consequently, for sustaining functional mitochondrial networks [21]. The fusion of mitochondria enhances the mixing of metabolites, proteins, and mtDNA, thereby preserving functional integrity and boosting ATP production efficiency. On the other hand, mitochondrial fission is primarily regulated by Drp1, which is recruited to the mitochondria via specific receptor proteins, including Fis1, MFF, and MiD49–51 [22]. Drp1 activity is modulated through post-translational modifications such as phosphorylation and SUMOylation, which promote the formation of helical oligomers that facilitate the cleavage of the mitochondrial membrane [23]. Fission is crucial for adapting to cellular energy demands, such as a decrease in ATP levels, by isolating damaged mitochondria. However, excessive fission may lead to fragmentation and disrupt the overall network homeostasis [24]. This dynamic balance between fission and fusion is finely tuned by external stimuli, including the cell’s energy status and apoptotic signals, ensuring that mitochondria remain adaptable and subject to quality control. Furthermore, the dynamics of mitochondrial morphology influence energy production, macromolecule synthesis, calcium signaling, redox balance, and metabolite signaling in response to cellular stress and nutritional fluctuations [25]. Notably, recent findings indicate that the mitochondrial import receptor TOM20 can directly interact with FEM1B—the substrate receptor of the cullin-RING E3 ubiquitin ligase 2 (CRL2)—to recruit the CRL2-FEM1B ubiquitin ligase complex [26]. This interaction regulates the turnover of PLD6, a key factor in mitochondrial dynamics, thereby influencing the delicate equilibrium of mitochondrial fission and fusion (Figure 1b).

### 2.3. Mitophagy

Mitophagy is an essential process that selectively identifies and removes damaged mitochondria through lysosomal degradation. This mechanism can be categorized into ubiquitin-dependent and ubiquitin-independent pathways. Initially, mitochondrial fission separates the damaged mitochondria from the healthy mitochondrial network, which are subsequently targeted for degradation via autophagy. In the ubiquitin-dependent pathway, a decrease in mitochondrial membrane potential (ΔΨm) leads to the accumulation of PINK1 on the outer mitochondrial membrane. This event activates Parkin, an E3 ubiquitin ligase, which subsequently ubiquitinates the impaired mitochondria [27]. Recent research has shown that mitochondrial reactive oxygen species (mtROS) function as signaling molecules that activate the ATM-CHK2 pathway. When activated, checkpoint kinase 2 (CHK2) interacts with ATAD3A, a protein in the mitochondrial membrane, phosphorylating it at Ser371 [28]. This phosphorylation inhibits the binding of PINK1 to the mitochondrial translocase complex (TOM22/TIM23), preventing its import into the inner mitochondrial membrane (IMM). As a result, PINK1 accumulates on the outer mitochondrial membrane (OMM) and initiates PINK1/Parkin-mediated mitophagy [28]. The ubiquitination of damaged mitochondria facilitates the recruitment of autophagy adaptors, such as P62 and OPTN, which bind to LC3, promoting the formation of autophagosomes. Conversely, the deubiquitinating enzyme USP30 modulates the intensity of mitophagy by preventing excessive clearance through deubiquitination, thus ensuring a balanced response to mitochondrial quality control [29]. Notably, a recent study reveals that cytosolic acetyl–coenzyme A (AcCoA) acts as a signaling metabolite to directly control mitophagy: when AcCoA levels decrease (e.g., during short-term fasting), the mitophagy receptor NLRX1 relieves autoinhibition and initiates mitophagy. This mechanism is independent of the canonical AMPK/mTOR pathways, offering a novel perspective into MQC [30]. The ubiquitin-independent pathway initiates mitophagy through direct binding of mitochondrial outer membrane receptors (BNIP3/NIX, FUNDC1, PHB2) or AMBRA1 to LC3/GABARAP [31]. In addition, other regulators, such as SMURF1, enhance mitophagic efficiency through ubiquitination. Mitophagy and MB exist in dynamic equilibrium, jointly maintaining energy metabolism and redox homeostasis [32] (Figure 1c).

In addition to classical pathways, non-classical mitochondrial clearance mechanisms (e.g., mitochondria-derived vesicles, transcellular mitophagy) have been reported, though their relevance to chemotherapy-induced toxicity remains unclear. On one hand, the PINK1/Parkin pathway lays the groundwork for autophagy by enhancing DrP1-mediated fission and inhibiting MFN1/2-mediated fusion. On the other hand, AMPK can activate PGC–1α, which in turn enhances the transcription of NRF1 and NRF2, thereby promoting FUNDC1-mediated mitophagy [33]. Additionally, AMPK activates autophagosome formation via ULK1 (Unc–51-like autophagy activating kinase 1) and directly phosphorylates mitochondrial fission factors to support the execution of the PINK1–Parkin pathway [34]. It is noteworthy that mitophagy and migrasome-mediated mitocytosis form a complementary clearance network, which constitutes a key mechanism for cells to maintain the quality and functional homeostasis of their mitochondrial population in response to mitochondrial damage of varying nature and severity [35].

### 2.4. Mitochondrial Proteostasis

Mitochondrial proteostasis is a fundamental module of the MQC system that preserves mitochondrial functional integrity by coordinating protein synthesis, folding, and degradation through molecular chaperones (HSP60/HSP10, HSP70), proteases (LONP1, ClpXP), and the mitochondrial unfolded protein response (UPRmt) [36,37]. In the molecular chaperone system, HSP60/HSP10 form an ATP-dependent “cage structure” to facilitate the refolding of unfolded proteins, while mtHSP70 stabilizes peptides transported across membranes [38,39]. The protease system plays a crucial role in the selective degradation of proteins through two main components: LONP1, which targets oxidatively damaged proteins, and ClpXP, which removes unassembled protein subunits [40]. The mitochondrial UPRmt is activated when there is an accumulation of misfolded proteins. During this process, CLPP-mediated cleavage generates short peptides that are released into the cytoplasm, where they activate the HSF1-ATF5 axis and the AMPK/mTORC1 signaling pathway. These pathways work together to regulate the expression of protective genes in the nucleus [41] (Figure 1d). In addition to their nuclear effects, the released short peptides interact with signaling molecules in the cytoplasm, further stimulating the nuclear translocation of transcription factors such as ATF4. This translocation promotes the expression of protective genes associated with amino acid metabolism and responses to oxidative stress. Consequently, these mechanisms work in concert to restore mitochondrial proteostasis and function through multiple pathways. By eliminating abnormal proteins, this system prevents metabolic dysfunctions such as oxidative phosphorylation (OXPHOS) failure and apoptosis, thus serving as a central mechanism for sustaining cellular energy homeostasis and stress resistance [42]. A conserved nutrient-responsive pathway (e.g., leucine–GCN2–SEL1L) also regulates mitochondrial proteostasis by stabilizing outer mitochondrial membrane proteins, though its role in chemotherapy-induced dysfunction remains to be clarified [43]. The traditional chaperone–protease–UPRmt network and the nutrient-responsive pathway (e.g., leucine–GCN2–SEL1L) synergistically eliminate abnormal proteins and stabilize functional proteins, preventing OXPHOS impairment and apoptosis to sustain cellular energy homeostasis and stress resistance. When proteostatic repair fails, mildly damaged mitochondria are shuttled to migrasomes for extracellular excretion (mitocytosis).

### 2.5. Migrasome-Mediated Mitocytosis: An Autophagy-Independent MQC Pathway

The core of MQC is the timely clearance of dysfunctional mitochondria to maintain the homeostasis of the intracellular mitochondrial network. Existing studies have clearly confirmed that “mitophagy” is a key pathway for the intracellular degradation of damaged mitochondria relying on lysosomes; however, a series of studies by Yu Li’s team from Tsinghua University have identified a novel vesicular organelle, the migrasome, and its mediated “mitocytosis”, which provides an autophagy-independent new mechanism of “extracellular excretion” for the MQC system [44].

The migrasome was first identified by Yu Li’s team in *Cell Research* in 2014, with an average diameter of approximately 2 μm. It is mainly formed by the detachment of the tips of cytoplasmic protrusions in migrating cells [45]. The core association between this organelle and mitochondrial management was further revealed by the team’s study published in *Cell* in 2021: when cells are exposed to mild mitochondrial stress (e.g., low-level oxidative damage induced by chemotherapeutic drugs), damaged mitochondria are directionally transported to migrasomes via the microtubule network [46]; subsequently, migrasomes detach from the mother cell and are released into the extracellular environment, physically excreting the damaged mitochondria (Figure 1e). This process is defined as mitocytosis. Specifically, TSPAN9 knockout mice exhibit impaired mitocytosis in bone marrow-derived macrophages (BMDMs) and peritoneal macrophages, leading to reduced MMP and compromised mitochondrial function [8].

Mitocytosis differs fundamentally from mitophagy: mitophagy degrades damaged mitochondria intracellularly via lysosomes, whereas mitocytosis achieves extracellular excretion through migrasomes [8]. Together, they form a coordinated quality control network of “intracellular degradation–extracellular excretion” [47]. When chemotherapeutic drugs such as anthracyclines and platinum-based agents induce mitochondrial damage at a rate exceeding the degradation capacity of mitophagy, mitocytosis can act as a “backup pathway” to reduce the intracellular accumulation of damaged mitochondria, preventing excessive release of ROS and triggering of cell apoptosis, thereby alleviating the toxicity of chemotherapy to highly metabolically active organs such as the heart, nerves, and kidneys.

Mitochondrial transport to migrasomes is characterized by a selective process: only mildly damaged mitochondria are recruited, while highly damaged ones are eliminated through mitophagy [45]. This selectivity is critical for preserving functionally normal mitochondria within cells, thereby preventing a reduction in mitochondrial quantity that could arise from the excessive degradation of mildly impaired mitochondria [8]. Such balance is essential for maintaining cellular energy metabolism and homeostasis, especially following chemotherapy. Subsequent studies have further found that the formation of migrasomes depends on actin-dependent motor proteins (e.g., Myosin–5a), which provides a potential molecular target for enhancing MQC function by regulating mitocytosis [46].

## 3. Role of MQC Dysregulation in Chemotherapy Toxicities

The widespread clinical use of chemotherapeutic agents has significantly improved tumor prognosis, yet their dose-limiting toxicities, such as cardiac injury, substantially restrict clinical benefits. Chemotherapeutic drugs disrupt key MQC regulatory mechanisms, including biogenesis, dynamics, autophagy, and proteostasis, causing multifold mitochondrial damage. Ultimately, this mitochondrial dysfunction leads to impaired function in metabolically active tissues. These mechanisms represent critical targets for understanding the underlying causes of toxic side effects and developing targeted protective strategies. Dysregulation of the core MQC regulatory axis (SIRT1/SIRT3–PGC–1α) is the “upstream switch” of DOX-induced cardiotoxicity, which forms a multidimensional damage network by crosstalking oxidative stress, mitochondrial dynamics, iron death, and inflammation in the tumor microenvironment. Targeting this axis can synchronize the direct and indirect damage pathways.

### 3.1. DOX-Induced Cardiac Injury

Doxorubicin (DOX) is a widely used anthracycline chemotherapeutic agent in clinical practice. However, DOX-induced cardiotoxicity (DIC) severely damages cardiomyocytes; impairs cardiac function, representing a key factor limiting its long-term use in cancer patients; and has become a major cause of cardiovascular complications in cancer survivors. Given that MQC may serve as a potential therapeutic target to address DOX-induced cardiotoxicity [48], it is crucial to deeply elucidate the underlying regulatory mechanisms of DIC and identify novel therapeutic targets to improve patient prognosis and advance clinical translation.

#### 3.1.1. Oxidative Stress

The mechanisms underlying DOX-induced cardiotoxicity are complex and multifaceted, with oxidative stress and mitochondrial dysfunction in cardiomyocytes serving as key initiating triggers for MQC disruption [49]. DOX significantly increases oxidative stress by disturbing the balance between reactive oxygen species (ROS) production and antioxidant defense systems [50]. Cardiomyocytes are particularly susceptible to oxidative damage due to their comparatively lower antioxidant capacity, including reduced expression of crucial antioxidant enzymes such as glutamate–cysteine ligase (GCLC), which is the rate-limiting enzyme in glutathione synthesis [51]. Importantly, the ROS generated by DOX is not confined to mitochondria; the NADPH oxidase (NOX) family, especially NOX2, also plays a significant role in ROS production [52]. DOX treatment upregulates TLR5 expression in cardiomyocytes. TLR5 directly interacts with Syk and is required for its full phosphorylation upon DOX stimulation. Phosphorylated Syk in turn activates PLCγ1, triggering downstream signaling events that ultimately induce the translocation of PKCα to the plasma membrane. Activated PKCα facilitates the translocation of p47phox, a key regulatory subunit of NOX2, from the cytoplasm to the plasma membrane, thereby driving the assembly of the active NOX2 complex and the massive production of superoxide anions, which is a central mechanism of oxidative damage in this acute cardiotoxicity model. Given that genetic deficiency or antibody-mediated neutralization of TLR5 potently attenuates cardiac injury in mice, targeting the TLR5 pathway emerges as a feasible therapeutic strategy against DOX-induced cardiomyopathy [53].

In cardiomyocytes, DOX induces extensive mitochondrial injury. Initially, DOX accumulates in the mitochondrial inner membrane by binding specifically to cardiolipin, which inhibits the activity of electron transport chain (ETC) complexes I, III, and IV [54]. This inhibition leads to an excessive generation of superoxide anions (O_2_^−^) and reactive nitrogen species (RNS), creating a damaging feedback loop characterized by “ETC inhibition → mtDNA damage → further deterioration of ETC function” [55]. Additionally, DOX interacts with mtDNA and topoisomerase 2β (Top2β) to form a ternary adduct of DOX, DNA, and Top2β, which significantly impairs oxidative phosphorylation (OXPHOS) by downregulating master regulators such as PGC-1α and PGC-1β [56]. Notably, DOX exerts its antitumor effect primarily by inhibiting Top2α in rapidly dividing cancer cells, while its dose-limiting cardiotoxicity results from the inhibition of Top2β, the predominant isoform expressed in post-mitotic cardiomyocytes. This tissue-specific expression of Top2 isoforms underlies the mechanistic divergence between its therapeutic and toxic effects [57]. The peroxidation of cardiolipin induced by DOX not only compromises mitochondrial membrane integrity but also converts cytochrome c from its role in electron transport to a peroxidase, promoting mitochondrial outer membrane permeabilization (MOMP), the release of pro-apoptotic factors, and the activation of caspase-dependent apoptosis pathways [57]. In addition, DOX–cardiolipin binding facilitates aberrant mitochondrial permeability transition pore (mPTP) opening, exacerbating mtDNA fragmentation and ETC subunit sulfhydryl oxidation. Collectively, these events severely impair ATP synthesis, leading to energy depletion in cardiomyocytes [58]. Oxidative stress further influences downstream pathological processes through various mechanisms. For instance, it exacerbates mitochondrial dynamics imbalance by modifying serine 616 on Drp1, a key regulator of fission [59]. Moreover, oxidative stress disrupts iron homeostasis by inhibiting the expression of the mitochondrial iron exporter ABCB8 while promoting the transcription of iron uptake-related proteins. This imbalance can lead to iron overload, which sets the stage for the initiation of ferroptosis [60].

In terms of cellular compensatory responses, although DOX activates the Keap1/Nrf2/ARE signaling pathway, resulting in the upregulation of antioxidant enzymes like manganese superoxide dismutase (MnSOD), cardiomyocytes have limited intrinsic antioxidant reserves [61]. Additionally, the time lag in transcriptional and translational responses hampers their ability to adequately counteract rapid surges in ROS [62]. Consequently, these intertwined processes lead to persistent mitochondrial respiratory dysfunction, excessive ROS accumulation, and increased cardiomyocyte apoptosis, ultimately contributing to cardiac functional deterioration [52]. This inadequate compensatory response is particularly pronounced in pediatric patients, representing a significant factor in their increased susceptibility to delayed cardiotoxicity [63].

#### 3.1.2. MB

**Figure 2:**
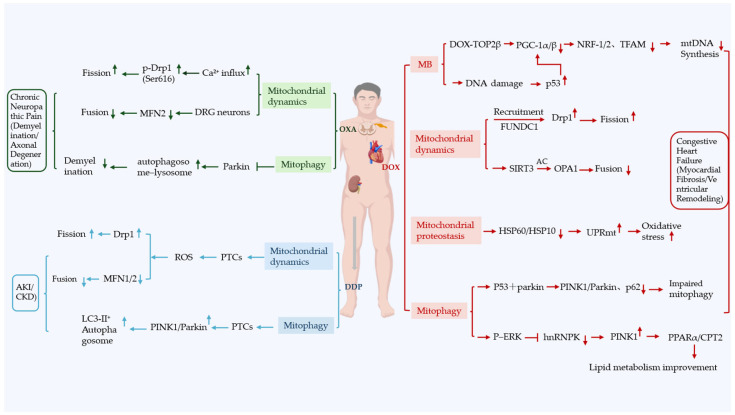
Mechanistic integration of chemotherapy-induced organ toxicity and MQC dysregulation. This diagram illustrates how three chemotherapeutic agents—doxorubicin (DOX), oxaliplatin (OXA), and cisplatin (DDP)—induce cardiac, neural, and renal toxicity by disrupting key mitochondrial quality control (MQC) modules, including mitochondrial dynamics, mitophagy, proteostasis, and biogenesis. DOX-induced cardiac toxicity (red pathway): DOX impairs mitochondrial biogenesis via TOP2β-PGC-1α/β axis inhibition, triggers mitochondrial dynamics imbalance (fission ↑, fusion ↓), suppresses mitophagy, and disrupts proteostasis, leading to congestive heart failure. OXA-induced neurotoxicity (green pathway): OXA induces Ca^2+^ influx in DRG neurons to activate Drp1-mediated fission, downregulates MFN2 to inhibit fusion, and represses mitophagy, resulting in chronic neuropathic pain and demyelination. DDP-induced renal toxicity (blue pathway): DDP promotes ROS production in PTCs to drive mitochondrial fragmentation, dysregulates mitophagy, and exacerbates oxidative stress, causing acute kidney injury (AKI) and chronic kidney disease (CKD). Abbreviations: DOX, doxorubicin; OXA, oxaliplatin; DDP, cisplatin. Arrow definitions: ↑: upregulation of molecular expression/activity, pathway activation, or enhancement of physiological processes; ↓: downregulation of molecular expression/activity, pathway inhibition, or attenuation of physiological processes; →: signal transduction, molecular interaction, or causal association; ⊣: direct inhibition of molecular expression/activity, pathway suppression, or attenuation of physiological processes.

Numerous studies indicate that DOX cardiotoxicity involves the disruption of mitochondrial biogenesis. In DOX-induced myocardial injury, the suppression of MB primarily stems from the interference with the transcriptional pathways regulated by PGC-1α and PGC-1β. DOX binds to cardiac-enriched TOP2β to form a DOX-TOP2β complex, which aberrantly binds to the gene promoters of PGC-1α and PGC-1β [64]. This complex physically blocks the binding of the PGC-1α transcription factor to the promoters of its downstream target genes, such as NRF1 and TFAM, thereby inhibiting their transcriptional activation. Additionally, by forming the topoisomerase IIβ–DOX–DNA cleavage complex, DOX induces DNA double-strand breaks [65]. This DNA damage leads to p53 activation. Activated p53 subsequently binds to and represses the promoters of PGC-1α and PGC-1β, achieving comprehensive suppression of the PGC-1α/β axis. This ultimately results in reduced expression of downstream related proteins, including NRF1, NRF2, and TFAM, thereby inhibiting mitochondrial biogenesis [64]. Consequently, these disruptions lead to severe energy metabolism dysfunction within cardiac tissue (Figure 2).

Notably, the SIRT1-PGC-1α signaling pathway can help mitigate this damage by adapting to energetic stress. Activation of SIRT1 promotes the deacetylation of PGC-1α, counteracting DOX-induced transcriptional repression and restoring NRF-1/TFAM-mediated MB and OXPHOS capacity. SIRT1 activators like resveratrol and SRT1720 significantly alleviate DOX-induced mitochondrial dysfunction in cardiomyocytes by enhancing the deacetylation state of PGC-1α [66]. In addition to SIRT1, mitochondrial-localized SIRT3 plays a crucial role in cardioprotection through various mechanisms. SIRT3 enhances antioxidant defense by activating mitochondrial enzymes such as MnSOD and isocitrate dehydrogenase 2 (IDH2), while also facilitating DNA repair by maintaining the activity of the oxidative DNA damage repair enzyme OGG1 [67]. Research has shown that SIRT3 increases the DNA-binding capacity of OGG1 through deacetylation, thereby improving the repair efficiency of 8-oxoguanine (8-oxoG) lesions and preserving mtDNA integrity [68]. This mechanism is vital in countering DOX-induced mtDNA mutations and the resulting dysfunction of the ETC. Furthermore, SIRT1 and SIRT3 work synergistically: SIRT3-mediated deacetylation of the FoxO1 transcription factor enhances MnSOD expression, while SIRT1 collaborates with the AMPK-PGC-1α axis to bolster MB and antioxidant defenses [69]. Given this synergistic functionality, therapeutic strategies targeting this signaling axis—such as combining NAD^+^ precursors with the SIRT3 activator honokiol—may effectively reduce DOX-induced cardiotoxicity. Such interventions not only promote MB via the SIRT1-PGC-1α pathway but also preserve mitochondrial functional integrity through SIRT3-mediated antioxidant and DNA repair mechanisms [70]. Additionally, alternative strategies, including enhancing PPARα expression or utilizing ferredoxin FGL, can independently elevate PGC-1α activity outside of the SIRT1/SIRT3 axis, further underscoring the critical role of compromised MB in DOX cardiotoxicity [71].

#### 3.1.3. Mitochondrial Dynamics

Mitochondrial dynamics, crucial for maintaining mitochondrial structural integrity, represent another key aspect of the MQC system. DOX-induced mitochondrial biogenesis dysfunction and oxidative stress can together trigger an imbalance in mitochondrial dynamics, further exacerbating MQC disruption and myocardial injury [72]. Specifically, disturbances in mitochondrial dynamics serve as a core pathophysiological mechanism underlying DOX-induced cardiotoxicity, with morphological imbalances closely linked to clinical cardiac dysfunction and delayed injury [73]. Research indicates that DOX has a bidirectional effect on proteins regulating mitochondrial morphology. It significantly reduces the expression levels of the fusion-associated proteins Mfn1, Mfn2, and OPA1 while simultaneously increasing the levels of the fission-associated protein Drp1 and Fis-1 [48]. This imbalance disrupts mitochondrial network integrity, leading to fragmented mitochondria, altered cristae architecture, and decreased cristae density. Collectively, these changes precipitate myocardial energy metabolism dysfunction, ultimately manifesting as clinically detectable reductions in left ventricular ejection fraction (LVEF) and elevations in cardiac troponin I/T (cTnI/T) [74]. At the molecular level, DOX-induced Drp1 activation is controlled by at least two routes. First, DOX-evoked ROS oxidize cardiolipin and activate PKCδ in cardiomyocytes [75]; however, the downstream step “PKCδ directly phosphorylates Drp1-Ser616” has so far been demonstrated only in hypoxia/reoxygenation or neuronal injury models, and remains to be verified under DOX treatment [76]. Second, DOX increases Drp1 accumulation on mitochondrial membranes via FUNDC1-mediated recruitment, further reinforcing mitochondrial hyper-fragmentation through the Drp1/FUNDC1 axis [77]. Notably, DOX exposure exerts dual inhibitory effects on OPA1, a protein critical for cristae stability, by reducing its expression levels and increasing acetylation-mediated modifications. These alterations impair the GTPase activity of OPA1, ultimately limiting mitochondrial fusion capabilities [78]. This DOX-induced OPA1 acetylation is mediated by the downregulation of SIRT3 (a mitochondrial deacetylase), creating a synergistic loop of “SIRT3 inhibition → OPA1 acetylation → fusion impairment → mitochondrial fragmentation” [79] (Figure 2). This mechanism has been linked to delayed cardiotoxicity, as evidenced by myocardial biopsy samples from pediatric leukemia patients following chemotherapy [79]. Of particular clinical relevance, disturbed mitochondrial dynamics interact synergistically with other key pathological mechanisms: fragmented mitochondria from dynamic imbalances not only directly impair oxidative phosphorylation efficiency but also exacerbate ferroptosis by releasing free iron ions and ROS [80]. Importantly, the use of the ferroptosis inhibitor Fer-1 or the mitochondria-specific antioxidant MitoTEMPO has been shown to alleviate both mitochondrial dynamic disorders and myocardial injury, providing novel insights for clinical combination interventions [81]. Existing intervention studies have begun to validate the clinical applicability of targeting mitochondrial dynamics. The Drp1-specific inhibitor Mdivi-1 has effectively corrected mitochondrial dynamic imbalances by reducing Drp1 and FUNDC1 expression while restoring OPA1 levels [79]. Moreover, overexpression of Mfn2 has been demonstrated to significantly mitigate cardiomyocyte apoptosis by enhancing mitochondrial fusion [82]. Genetic analyses further support these findings; heterozygous deletion of Drp1 markedly attenuated DOX-induced mitochondrial fragmentation and cardiac dysfunction, reinforcing the notion that Drp1 inhibitors may present a promising preventive strategy for high-risk populations, including children and individuals with a history of heart disease [83].

#### 3.1.4. Mitophagy

It is established that dysregulated mitophagy, induced by DOX in cardiomyocytes, results in the disruption of ATP synthesis, the opening of mPTPs, and consequent cell death [64]. Central regulatory pathways particularly involve two key molecular axes: PINK1/Parkin and Bcl-2/Bnip3 [84]. Notably, experimental models of acute high-dose DOX administration—which serve as a critical paradigm to distinguish pathological mitochondrial over-clearance from adaptive protective mitophagy—have uncovered a striking dose-dependent and paradoxical role of Parkin in doxorubicin-induced cardiotoxicity. In support of this, a pivotal study employing H9c2 cardiac myoblast cells under acute high-dose DOX treatment demonstrated that Parkin knockdown significantly attenuated cardiomyocyte death, whereas Parkin overexpression exacerbated it, directly implicating Parkin-mediated mitophagy in the detrimental over-clearance of mitochondria [83]. This study, along with supporting in vivo evidence, establishes that under conditions of severe, acute insult, hyperactivated Parkin-mediated mitophagy contributes to cardiac injury. The mechanistic basis for this dual role is thus understood to hinge on the magnitude of DOX-induced mitochondrial damage: whereas protective mitophagy is elicited under conditions of moderate damage, the present findings delineate the specific pathway—unchecked DRP1 fission leading to Parkin-dependent mitophagic hyperactivation—that underlies the pathological “over-clearance” observed under acute high-dose stress. This outcome directly links the functional consequence of Parkin-mediated mitophagy to the intensity of DOX-induced stress [85]. Notably, mitophagy dysfunction amplifies other MQC impairments: unresolved fragmented mitochondria induce proteostatic overload via misfolded protein release, while their ROS production further suppresses PGC-1α/β to exacerbate biogenesis failure [86,87]. Research has shown that DOX activates a cascade through p53, whereby activated p53 directly interacts with Parkin to form a complex that retains Parkin in the cytoplasm. This retention prevents Parkin from translocating to damaged mitochondria, leading to a significant reduction in the expression of mitochondrial-localized PINK1, Parkin, and the autophagy adaptor protein p62 [85]. This p53-mediated inhibition forms a dual hit on MQC, simultaneously blocking mitophagy and repressing PGC-1α/β transcription to impair biogenesis [85]. Consequently, this impaired mitochondrial targeting results in inefficient clearance of dysfunctional mitochondria, ultimately leading to cardiomyocyte death. In addition to cardiomyocytes, DOX can also induce a p53–Parkin axis-mediated specific blockage of mitophagy in CFs, which serves as a crucial non-cardiomyocyte mechanism underlying its induction of MQC dysfunction. DOX triggers DNA damage in CFs and elevates p53 expression by threefold; the activated p53 binds and sequesters Parkin, thereby significantly inhibiting the translocation of Parkin to damaged mitochondria, and the interaction between these two proteins is markedly enhanced following DOX treatment. In contrast, p53 knockout restores the mitochondrial localization of Parkin and the normal mitophagic process in CFs, while simultaneously reversing DOX-induced functional abnormalities in CFs, including impaired proliferation and migration, excessive production of mitochondrial ROS, and mitochondrial membrane potential depolarization. This mechanism impairs the clearance of damaged mitochondria in CFs, which further exacerbates oxidative stress and pathological remodeling of the myocardial microenvironment, and drives the progression of DOX-induced cardiotoxicity [88].

In addition to p53-mediated regulation of Parkin, recent studies have identified heterogeneous nuclear ribonucleoprotein K (hnRNPK) as a key upstream regulator of the PINK1/Parkin pathway, demonstrating a protective role in DOX-induced cardiotoxicity. DOX activates P–ERK to both inhibit hnRNPK expression and promote its cytoplasmic translocation; in contrast, hnRNPK overexpression improves cardiac function, alleviates myocardial injury, and reverses lipid metabolic dysregulation by restoring PPARα/CPT2 expression [89]. hnRNPK sequentially couples mitochondrial quality control to lipid homeostasis: it first transcriptionally upregulates PINK1, thereby activating PINK1/Parkin-mediated mitophagy; this mitophagic flux then acts as an obligatory upstream event that restores PPARα/CPT2 expression and rescues lipid metabolism. Consequently, the metabolic benefits are contingent upon—rather than parallel to—the prior enhancement of mitochondrial autophagy [89]. This mitophagic activation is characterized by an increase in LC3-II levels, a decrease in P62 expression and reactive oxygen species (ROS) production, and a restoration of mitochondrial morphology and function, which is a prerequisite for the subsequent recovery of PPARα/CPT2-mediated fatty acid β-oxidation and reversal of lipid metabolic dysregulation—as evidenced by the abrogation of hnRNPK-induced PPARα/CPT2 upregulation and lipid accumulation reduction upon treatment with the mitophagy inhibitor Mdivi-1 [89]. However, the protective effects of this pathway can be negated by mitophagy inhibitors, confirming the indispensability of mitophagy in hnRNPK’s cardioprotective cascade. Importantly, hnRNPK does not influence Parkin levels but specifically targets and regulates PINK1, thereby enriching the upstream transcriptional regulatory network of the PINK1/Parkin pathway. This presents a novel strategy for restoring mitophagic homeostasis by targeting hnRNPK or inhibiting phosphorylated ERK (P-ERK) [89] (Figure 2).

Central regulatory pathways follow a hierarchical order: primary control is exerted by the PINK1–Parkin axis (fine-tuned by p53 or hnRNPK), and—when this core system is overwhelmed—secondary/compensatory rescue is provided by the Bcl-2/Bnip3/Nix module [90]. Bnip3-mediated mitophagy is tightly intertwined with mitochondrial dynamics: SIRT3 downregulation by DOX not only impairs fusion via OPA1 acetylation but also upregulates Bnip3, and their imbalance further disrupts mitochondrial networks [91]. Additionally, as a secondary compensatory response to impaired primary mitophagy, mitochondrial outer membrane proteins Bnip3 and Nix act as autophagy receptors that mediate mitophagy by linking damaged mitochondria to autophagosomes via LC3 binding. Research indicates that DOX exposure elevates Bnip3 expression and facilitates its translocation to mitochondria, thereby enhancing mPTP permeability and causing mitochondrial membrane depolarization [92]. Experimental interventions have shown cardioprotective effects following either the pharmacological inhibition of Bnip3 or genetic modifications using Bnip3 mutant constructs [93]. Furthermore, SIRT3 mitigates DOX-induced injury by suppressing Bnip3 expression; however, paradoxically, its overexpression can exacerbate cellular injury, emphasizing the importance of finely tuned, dose-dependent regulation within this signaling pathway [94]. While the precise molecular mechanisms by which DOX activates Bnip3 are not fully understood, oxidative stress mediated by ROS likely plays a significant role in this activation [95]. In addition, Nix works synergistically with Bnip3 to promote cardiomyocyte apoptosis and mitophagy, underscoring the critical involvement of mitochondrial autophagy receptors in the pathogenesis of DOX-induced cardiac injury [96]. Collectively, disrupting p53–Parkin, cardiac-targeted hnRNPK modulation and Bnip3 inhibition all exhibit preclinical cardioprotective feasibility in DIC, with hnRNPK (via AAV) and Bnip3 inhibition showing relative cardiac specificity whereas p53–Parkin disruption carries off-target risks [97]; key translational barriers include the need for fine-tuned dose regulation, lack of clinical-grade delivery systems and the absence of non-human primate validation for these approaches.

#### 3.1.5. Mitochondrial Proteostasis

Mitochondrial proteostasis is a critical aspect of the MQC system, responsible for maintaining the proper folding, transport, and degradation of mitochondrial proteins. This balance is disrupted by DOX-induced oxidative stress and mtDNA damage, which in turn impairs the function of ETC complexes, mitochondrial biogenesis regulators (e.g., PGC-1α), and dynamics-related proteins (e.g., OPA1), thereby exacerbating MQC disruption [98]. Mitophagy dysfunction further aggravates proteostatic overload by failing to clear damaged mitochondria that release misfolded polypeptides, exceeding the capacity of chaperones (e.g., HSP60/HSP10) and proteases (e.g., LonP1) [87,99,100,101].

Human mitochondria contain over a thousand proteins, but only 13 components of the ETC are encoded by mtDNA [102]. The remaining proteins are encoded by nDNA, synthesized by cytoplasmic ribosomes, and subsequently transported to specific mitochondrial compartments, such as the outer mitochondrial membrane (OMM) or the mitochondrial matrix, via translocator of the inner membrane (TIM) and translocator of the outer membrane (TOM) complexes. Proper localization of these proteins relies on mitochondrial targeting sequences (MTSs), which are cleaved by matrix-resident peptidases after transportation, ensuring functional specificity [103]. Under pathological stress conditions like DOX-induced cardiotoxicity, mitochondrial proteostasis is regulated through molecular chaperones, proteolytic enzymes, and UPRmt [104]. Among the chaperone systems, the HSP60/HSP10 (localized in the mitochondrial matrix of cardiomyocytes) complex facilitates proper protein folding within a cage-like structure; impairment of this complex can trigger UPRmt activation and excessive ROS production, ultimately leading to cardiomyocyte apoptosis [105] (Figure 2). Elevated expression of the HSP60/HSP10 complex stabilizes mitochondrial function through dual mechanisms: increasing the levels of anti-apoptotic proteins (such as Bcl-xL and Bcl-2) while suppressing pro-apoptotic proteins like Bax and inhibiting the ubiquitination of Bcl-xL [56]. SP70 expression also increases during cardiac injury; neutralizing antibodies against HSP70 have been shown to reduce cardiac fibrosis by inhibiting TLR2-NF-κB-mediated inflammatory pathways [106]. Furthermore, exercise-induced cardioprotection is linked to increased HSP60 expression and elevated levels of glutathione (GSH), which enhance myocardial antioxidant defenses, presenting a promising therapeutic avenue [56]. In the proteolytic system, downregulation of LonP1 (a matrix-resident ATP-dependent protease highly expressed in cardiomyocytes) leads to abnormal protein accumulation, accelerating the progression toward heart failure (HF) [107]. Additionally, the proteases YME1L1 and OMA1 oversee the cleavage of the mitochondrial fusion protein OPA1. An imbalance in this proteolytic axis, as seen in YME1L1 deficiency, disrupts the long-to-short isoform ratio of OPA1, resulting in mitochondrial fragmentation—a hallmark of cardiac dysfunction and myocardial injury [56]. Mitochondria also respond to stress via retrograde signaling pathways, including nuclear ATF4/ATF5–CHOP (which mitigates proteotoxic stress), Sirt3–FOXO3a–SOD2 (which enhances antioxidant capacity), and ERα–NRF1–HTRA2 (which promotes the clearance of misfolded proteins) [86]. These pathways work synergistically to upregulate mitochondrial chaperones and proteases, thereby restoring mitochondrial protein homeostasis. While these mechanisms exhibit cardioprotective effects in conditions such as myocardial infarction and HF, further studies are needed to fully elucidate their dynamic responses during cardiac injury, particularly their interactions with mitochondrial dynamics imbalances and mitophagy dysfunction [108].

In summary, the MQC dysfunction in doxorubicin-induced cardiotoxicity exhibits distinct cardiac specificity: the core driver is the binding of highly expressed TOP2β in cardiomyocytes to PGC-1α, which inhibits mitochondrial biogenesis. This primary defect initiates a cascade of interconnected MQC impairments—triggering dynamics imbalance, proteostatic overload, and mitophagy dysfunction that fails to clear damaged organelles, accompanied by abnormal transcellular mitophagy between cardiac resident macrophages (CRMs) and cardiomyocytes. This mechanism is inherently distinct from the MQC impairment pathways in the nervous system and kidneys.

### 3.2. Oxaliplatin-Induced Neurotoxicity

Chemotherapy-induced peripheral neuropathy (CIPN) is a common dose-limiting toxicity of conventional chemotherapeutic agents including platinum agents, taxanes, and vinca alkaloids, which severely compromises patients’ quality of life and forces dose reduction or even treatment discontinuation. Its unifying pathogenic core lies in cumulative mitochondrial injury in the sensory neurons of dorsal root ganglia (DRGs) [109]. Taking oxaliplatin as a typical example, this agent selectively accumulates in sensory neurons, inducing oxidative stress, deranging mitochondrial dynamics and impairing autophagic function, which ultimately leads to neuronal injury [110]. Clinically, it is characterized by a length-dependent glove-and-stocking pattern of sensory disturbances and classified into two forms: an acute reversible form and a chronic cumulative form. Both forms of neurotoxicity are underpinned by multidimensional dysfunction of the MQC system [111]. The following sections dissect these MQC defects in chronological order and explore the corresponding surveillance biomarkers and potential therapeutic nodes.

#### 3.2.1. Oxidative Stress

The pathogenesis of CIPN remains incompletely understood, with injury affecting multiple structures and pathways of the peripheral nervous system (PNS). Oxidative stress is one of the core pathogenic mechanisms, and is particularly critical in DRG sensory neurons [112].

The PNS is highly susceptible to oxidative damage due to distinct characteristics, including the lack of an effective blood–nerve barrier, insufficient lymphatic drainage, neurons enriched with phospholipid membranes and mitochondria, and limited endogenous antioxidant defenses [113]. The accumulation of platinum agents like oxaliplatin in DRG is a critical initial step in neuropathy development [114]. A key study has identified that this accumulation is primarily mediated by the organic cation transporter 2 (OCT2, encoded by SLC22A2), which is highly expressed not in sensory neurons themselves, but in the satellite glial cells that envelop them. This OCT2-mediated uptake into satellite glia triggers a cascade of events, including oxidative stress and neuronal hypersensitivity, ultimately leading to pain and neurotoxicity [115]. These agents covalently bind to DNA in DRG neurons, forming adducts while simultaneously inhibiting mitochondrial electron transport chain complexes I and III [116]. This disruption leads to an excessive production of superoxide anions that surpasses the scavenging capacity of superoxide dismutase (SOD), triggering both primary and secondary oxidative stress cascades [117]. Additionally, platinum compounds likely target mitochondrial DNA and respiratory-chain complexes in Schwann cells, increasing ROS and compromising bioenergetics; this sets up a detrimental feedback loop of mitochondrial damage → excessive ROS → energy dysfunction, which may predispose to demyelination. Elevated ROS in DRG neurons not only triggers apoptosis and energy failure but also induces IL-1β, IL-6, TNF, and CX3CL1 upregulation [118]. This contributes to a pro-inflammatory milieu that likely enhances the hyperexcitability of TRPV1^+^ DRG neurons [119]. Notably, oxaliplatin rapidly undergoes non-enzymatic conversion to oxalate in plasma [114]. Acting as a Ca^2+^ chelator, oxalate lowers extracellular Ca^2+^ and thereby increases neuronal excitability [120]; this Ca^2+^-dependent increase in excitability, together with oxalate-induced upregulation of TRPM8 channels via Ca^2+^-influx/NFAT signaling, contributes to acute cold hypersensitivity by enhancing TRPM8-mediated currents in small-diameter DRG neurons [121].

#### 3.2.2. Mitochondrial Dynamics

Like cisplatin, oxaliplatin has been reported to reduce MFN2 levels in DRG neurons, leading to impaired mitochondrial fusion and axonal transport (direct binding to the GTPase domain has yet to be demonstrated) [122]. Platinum-based chemotherapeutic agents, such as oxaliplatin, induce neurotoxicity primarily through their adverse effects on mitochondrial dynamics in DRG neuron axonal mitochondria [115]. In vivo studies employing rat spinal cord tissues have demonstrated that Ca^2+^/calmodulin-dependent protein kinase II (CaMKII) is among the first to be activated in response to oxaliplatin treatment. Oxaliplatin enters small-diameter DRG neurons via OCT1/2 transporters [115], and its metabolite oxalate chelates extracellular Ca^2+^ to trigger Ca^2+^ influx through TRPM8 channels; this elevated intracellular Ca^2+^ binds calmodulin to activate CaMKII [123], which directly phosphorylates Drp1 at Ser616. Oxaliplatin-induced Ca^2+^ influx (via TRPM8 channels) binds calmodulin, triggering CaMKII autophosphorylation at Thr286—a modification required for its kinase activity [123]. Inhibition of CaMKII using KN-93 effectively abolishes Drp1-S616 phosphorylation and the associated mechanical hypersensitivity, indicating that this activation is crucial for the phosphorylation of the Ser616 site on Drp1 in DRG neuron axonal mitochondria, thereby enhancing its GTPase activity [124] (Figure 2).

The resulting surplus of fragmented mitochondria further amplifies local stress: COX-2 is up-regulated and released from injured sensory terminals, activating the TLR4/NF-κB axis in spinal microglia and driving IL-6/TNF-α secretion [125]. Concurrently, oxaliplatin suppresses Mn-SOD activity, attenuating ROS clearance and establishing a self-amplifying loop of ROS → COX-2/PGE2 → inflammation → mitochondrial damage that culminates in energetic failure. Collectively, these findings identify excessive mitochondrial fission and impaired fusion as a central, dose-dependent driver of chronic OIPN [126].

#### 3.2.3. Mitophagy

While dynamics imbalance fragments mitochondria, oxaliplatin simultaneously paralyzes the organelle’s disposal system—mitophagy—in DRG neuron somata and axonal mitochondria, turning a self-repair program into a self-poisoning loop [114]. Oxaliplatin disrupts mitochondrial homeostasis by inducing a “self-eating but not self-digesting” phenotype—a hallmark of mitophagic flux blockage in neurodegenerative disorders [114]. The drug first inhibits respiratory complexes I/III, provoking superoxide bursts, membrane depolarization, and ATP drop. These stress signals stabilize PINK1 on the outer membrane, which in turn phosphorylates Parkin and licenses mitophagy (increased LC3-II, autophagosome number) [127,128]. However, this early recruitment is transient: notably, beyond the initial 6–12 h, oxaliplatin has been shown to directly suppress Parkin expression, switching the pathway from primed to paralyzed and thereby arresting mitophagic flux at an upstream level [129]. Oxaliplatin stalls autophagosome–lysosome fusion, giving rise to p62 aggregates, fewer autolysosomes, and prolonged retention of dysfunctional mitochondria that continuously release cytochrome-c and mtDNA [130,131] (Figure 2). Entrapped mitochondria are partly exported as mitochondria-derived vesicles, but this escape valve is insufficient, so ROS remain high and demyelination proceeds. This vicious cycle of mitophagic failure contributes to neuronal apoptosis, evidenced by shifts in the Bcl-2/Bax ratio and caspase-3 activation [129]. Restoring autophagic flux—by enhancing Parkin-mediated mitophagy with agents like salidroside—re-establishes autophagic flux, halves ROS levels and alleviates oxaliplatin-induced neuropathy in vivo [129].

#### 3.2.4. Research Gaps and Hypotheses in Chemotherapy-Induced Neurotoxicity

Integrating findings from Section 3.2.1, Section 3.2.2 and Section 3.2.3, oxaliplatin induces peripheral neuropathy in subtype-specific DRG neurons through a sequential disruption of core MQC functions: by activating oxidative stress, driving excessive mitochondrial fission, and blocking mitophagic flux. While these constitute the currently well-established core mechanisms, existing research primarily covers only three of the five MQC modules. The roles of MB and mitochondrial proteostasis remain in preliminary exploratory stages—particularly in DRG neuron axonal mitochondria and their accompanying Schwann cells [132]. Given the weak regenerative capacity and high dependence on mitochondrial energy of DRG sensory neurons, we hypothesized that oxaliplatin may impair neuronal energy supply and damage-repair capability by inhibiting PGC-1α-mediated mitochondrial biogenesis in DRG neuron somata. As one speculative extension, oxaliplatin-induced ROS has been proposed—though not yet evidenced—to trigger the p53/miR-34a axis in cancer cells; since this axis represses PGC-1α transcription in Parkinson’s disease models, we hypothesize (but do not demonstrate) that an analogous mechanism might operate in DRG neurons exposed to oxaliplatin. Intriguingly, the mitochondrial adenosine A3 receptor (A3AR) has emerged as a novel therapeutic target. Recent findings indicate that activation of A3AR can reverse the ATP decline induced by oxaliplatin, suggesting a potential strategy for restoring mitochondrial bioenergetics downstream of PGC-1α inhibition [133]. Additionally, the long-distance axonal transport characteristic of neurons can exacerbate the effects of proteostatic imbalance in axonal mitochondria. Misfolded proteins involved in axonal transport and the functioning of inner mitochondrial membrane complexes may disrupt mitochondrial distribution along axons, thereby intensifying neural damage [134]. This proteostatic challenge may also relate to dysregulation of UPRmt, a crucial stress-response signaling pathway known to be activated in various neurodegenerative conditions; however, its role in CIPN remains largely unexplored [135].

In conclusion, future research on chemotherapy-induced neurotoxicity should extend beyond the current focus on mitochondrial dynamics and mitophagy to encompass the entire MQC network, with particular emphasis on modules that are uniquely regulated in high-energy-demand DRG neuron subtypes. Moreover, studies should move beyond a neuron-autonomous perspective to explore innovative paradigms such as intercellular mitochondrial transfer from supportive cells (e.g., monocytes) to damaged neurons, representing a novel exogenous rescue mechanism for neuroprotection.

### 3.3. Cisplatin-Induced Renal Injury

Chemotherapeutic agents commonly used in cancer treatment can inflict specific damage on proximal tubular epithelial cells (PTCs)—the primary target of cisplatin nephrotoxicity—and disrupt the glomerular filtration barrier due to their nonselective cytotoxic effects and reliance on renal clearance [136]. Clinically, this toxicity is evidenced by a reduction in glomerular filtration rate (GFR), electrolyte imbalances, and the accumulation of metabolic waste products, which may necessitate dose adjustments or the discontinuation of chemotherapy [137]. Epidemiological studies indicate that cisplatin-induced AKI occurs in 20–70% of exposed patients, with the incidence rising to approximately 70% when cumulative doses exceed 300 mg/m^2^; acute tubular necrosis (ATN) is the predominant pathological manifestation [138]. Currently, clinical strategies employing antioxidants, anti-inflammatory agents, or anti-apoptotic therapies have limited success in alleviating renal dysfunction [139]. Established evidence identifies MQC imbalance as a central mechanism underlying chemotherapy-associated nephrotoxicity: cisplatin triggers mitochondrial cascade injury via excessive ROS production, leading to impaired OXPHOS, ΔΨm depolarization, disturbances in mitochondrial dynamics, and obstruction of PINK1/Parkin-mediated mitophagy [140], which sequentially converge on global MQC failure to drive cisplatin-induced renal toxicity.

#### 3.3.1. Oxidative Stress

Accumulating established evidence has clearly demonstrated that oxidative stress is closely linked to PTCs ferroptosis [141], serving as a core mechanism underlying cisplatin-induced acute kidney injury (AKI) and collectively driving the progression of nephrotoxicity [142]. As a clinically widely used chemotherapeutic agent for solid tumors, cisplatin’s dose-dependent nephrotoxicity severely limits its clinical application and represents one of the major causes of clinical AKI [143]. Ferroptosis, characterized by the accumulation of lipid peroxidation, has been confirmed as an established pathological process in cisplatin-induced renal cell injury [144]. Cisplatin significantly promotes the accumulation of ROS in renal tissues. On one hand, it directly disrupts the redox network, inducing damage to DNA, proteins, and lipids [145]. On the other hand, mitochondrial ROS in PTCs trigger SIRT3 SUMOylation, resulting in decreased SIRT3 expression, which in turn enhances mitochondrial dihydroorotate dehydrogenase (DHODH) acetylation and inhibits its function [146]. This exacerbates coenzyme QH2 (CoQH2) depletion, which, combined with cisplatin-induced downregulation of Nrf2 pathway components (e.g., HO-1) and the activity of antioxidant enzymes (such as SOD and GSH), amplifies the “ROS accumulation → lipid peroxidation” cascade. Meanwhile, cisplatin regulates acyl–CoA synthetase long-chain family member 4 (ACSL4) and lysophosphatidylcholine acyltransferase 3 (LPCAT3) to promote the synthesis of polyunsaturated fatty acid-containing phospholipids (PUFA–PLs) and inhibits the glutathione peroxidase 4 (GPX4)/ferroptosis suppressor protein 1 (FSP1)–CoQ10 defense systems, ultimately inducing renal cell ferroptosis [146].

These mechanisms collectively contribute to pathological features such as edema and necrosis of renal tubular epithelial cells, along with a decrease in mitochondrial density within cortical renal tubules [147]. Targeted interventions have demonstrated effectiveness in alleviating these effects. For instance, the mitochondria-targeted antioxidant mitoquinone (MitoQ) and the NAD^+^ precursor nicotinamide mononucleotide (NMN) can inhibit the acetylation of dihydroorotate dehydrogenase (DHODH), thereby preventing ferroptosis and acute kidney injury (AKI) [148]. Furthermore, overexpression of DHODH has been shown to mitigate CoQH2 depletion and lipid peroxidation [149], indicating its potential as a regulatory target in therapeutic strategies.

#### 3.3.2. Mitochondrial Dynamics

Cisplatin induces excessive mitochondrial fragmentation primarily in PTCs by ROS-dependent Drp1 activation and concurrent MFN1/2 suppression [150], consistent with the high mitochondrial content and energy dependence of PTCs. Cisplatin accumulates in renal proximal tubules via OCT2 transporters [151], and its aquated form binds to mtDNA to induce ROS production [152]. This process promotes Drp1 translocation to the mitochondrial outer membrane and stimulates the assembly of fission complexes, ultimately disrupting the integrity of the mitochondrial network [153]. This fragmented mitochondria show an increased tendency to release pro-apoptotic factors, such as cytochrome C, and exhibit diminished ATP synthesis capacity, thereby exacerbating apoptosis and functional impairment in renal tubular epithelial cells [150,154]. Research indicates that ROS generated by cisplatin not only enhance mitochondrial fission through oxidative modifications of Drp1 but also inhibit mitochondrial fusion by targeting fusion proteins like MFN1 and MFN2 [155]. Furthermore, cisplatin reduces the expression of MFN1 and MFN2, impairing the fusion-mediated mechanisms that are crucial for mtDNA repair and the distribution of antioxidants [156]. This imbalance between mitochondrial fusion and fission further intensifies mitochondrial fragmentation, creating a detrimental cycle of mitochondrial dysfunction (Figure 2). Thus, cisplatin disrupts mitochondrial dynamics primarily via the ROS/Drp1/MFN1/2 axis, concurrently acting with the inhibition of the PINK1/Parkin-mediated autophagic pathway [157]. This dual action leads to the accumulation of mitochondrial damage, impaired energy metabolism, and apoptosis, all of which contribute to renal tubular injury [154]. Targeted interventions aimed at modulating mitochondrial dynamics, such as Drp1 inhibitors (e.g., Mdivi-1) or activators of MFN2, as well as strategies to enhance autophagic flux, represent promising approaches to mitigate cisplatin-induced nephrotoxicity [158].

#### 3.3.3. Mitophagy

Established research indicates that cisplatin initially activates the PINK1–Parkin mitophagy pathway in PTCs, as evidenced by increased localization of PINK1 on mitochondrial membranes and the recruitment of Parkin, which promotes the formation of autophagosomes associated with LC3-II [159,160]. However, prolonged cisplatin exposure disrupts subsequent autophagosome–lysosome fusion, resulting in impaired autophagic flux and elevated levels of mitochondrial proteins such as TOM20, reflecting the accumulation of damaged mitochondria [160]. This mitophagic failure, a terminal step in cisplatin-induced MQC collapse, contributes to structural damage (including mitochondrial swelling and cristae rupture), leakage of mtDNA, activation of inflammatory pathways, and cytochrome c-mediated apoptosis, collectively exacerbating renal tubular epithelial injury [161] (Figure 2).

Beyond cisplatin, other chemotherapeutic drugs, including paclitaxel analogs (e.g., paclitaxel), antimetabolites (e.g., methotrexate, gemcitabine), and alkylating agents (e.g., cyclophosphamide), have also been implicated in nephrotoxicity, with emerging evidence supporting a similar link to MQC disruption [162]. Collectively, cisplatin sequentially oxidizes–fragments–entraps renal mitochondria, creating a ROS-Drp1-MFN2 feed-forward loop, culminating in mitophagic paralysis and tubule cell death as a result of global MQC failure [163]. Human organoid and early-phase trial data now validate targeting mitochondrial dynamics or NAD^+^ repletion as clinically translatable renoprotective strategies [160].

#### 3.3.4. Research Gaps and Hypotheses in Chemotherapy-Induced Nephrotoxicity

Current research on cisplatin-induced nephrotoxicity predominantly focuses on established MQC mechanisms such as oxidative stress and dynamic imbalance. However, from the perspective of the complete MQC network, significant knowledge gaps remain concerning the roles of MB, proteostasis, and the yet-to-be-validated migrasome-mediated mitocytosis “pressure-release valve” pathway in renal injury [164]. It is essential to ascertain whether cisplatin impairs the mitochondrial regenerative capacity and post-injury repair mechanisms of renal tubular epithelial cells by inhibiting the PGC-1α–NRF1/TFAM axis [165]. Furthermore, research is needed to investigate whether cisplatin-induced protein misfolding overloads the HSP60/LONP1 proteostasis system and to clarify the regulatory role of UPRmt in determining tubular cell fate (necrosis vs. survival). Additionally, it remains to be experimentally verified whether highly metabolic and polarized renal epithelial cells possess the migrasome-mediated mitocytosis machinery (potentially involving key molecules like TSPAN4) and whether cisplatin disrupts this function [166]. Future studies must systematically analyze the physiological and pathological dynamics of these MQC modules in the kidney to fill these cognitive gaps and identify novel targets for specific renoprotective strategies.

In particular, MQC dysfunction in cisplatin-induced nephrotoxicity exhibits distinct renal specificity: it primarily targets proximal tubular epithelial cells (PTECs), induces ferroptosis via ROS-mediated DHODH acetylation, and is associated with putative impairment of tetraspanin 4 (TSPAN4)-dependent mitocytotic function [146,166]. This shows significant differences from the clearance mechanisms in the heart (TSPAN9-dependent) and nervous system (with weak mitocytotic activity) [140,167]. These organ-specific MQC features, alongside their distinct expression patterns in normal vs. tumor cells, represent an emerging hypothesis that provides a molecular basis for dissociating anti-tumor efficacy from off-target organ toxicity via selective MQC modulation.

### 3.4. Brief Comparison of Organ-Specific MQC Dysregulation

Chemotherapeutic agents induce organ-specific MQC disruption due to differences in cell type, metabolic activity, drug accumulation, and other factors. The core characteristics of three typical toxicities are compared as follows (Table 1):

## 4. Intervention Strategies Targeting MQC to Mitigate Chemotherapy-Induced Side Effects

Mitochondrial dysfunction—manifesting as impaired bioenergetics, ΔΨm depolarization, and ROS overload—is a final common pathway of chemotherapy-induced cardiotoxicity, neurotoxicity, and nephrotoxicity, positioning mitochondria as high-value therapeutic targets. A growing repertoire of small-molecule compounds and natural products has been shown to restore specific MQC modules (biogenesis, dynamics, mitophagy, proteostasis, or mitocytosis) and to translate into organ protection in preclinical models (Table 2). All these interventions exert organ-protective effects by targeting the dysregulated core nodes of the MQC network, and their core therapeutic logic is to restore the structural and functional homeostasis of the MQC system—including activating defective mitochondrial biogenesis, reversing imbalanced mitochondrial dynamics, reactivating blocked mitophagic flux, or enhancing impaired mitocytosis—thus fundamentally blocking the cascade reaction of mitochondrial damage induced by chemotherapeutic agents and dissociating off-target organ toxicity from anti-tumor efficacy.

### 4.1. Small-Molecule Compounds

Advances in mitochondrial biology have progressively demonstrated that small-molecule compounds show considerable promise in precisely mitigating chemotherapy-associated toxicity by targeting pivotal MQC processes, including mitochondrial dynamics, mitophagy, and oxidative stress, and mitochondrial biogenesis. Fundamental studies have elucidated the mechanisms underlying these compounds (e.g., inhibition of Drp1, activation of SIRT1/AMPK), which directly repair MQC system disruption induced by chemotherapeutics [56]. Currently, such compounds possess distinct advantages such as precise targeting, favorable cellular permeability, and potential for drug repurposing [202]. Yet they face substantial clinical translation challenges, including off-target effects, suboptimal pharmacokinetic profiles (e.g., short in vivo half-life, poor bioavailability), and narrow therapeutic windows, despite promising preclinical efficacy. This underscores the urgent need to optimize their target specificity and develop mitochondria/organ-targeted delivery systems—an unmet need that is further amplified by the fact that chemotherapeutics (e.g., cisplatin, doxorubicin) exert antitumor effects via inhibiting cancer cell proliferation but trigger multi-organ toxicities primarily by inducing extensive MQC disruption. As precise modulators of cellular signaling networks, small-molecule compounds are pivotal for drug development due to their ability to dynamically regulate intracellular pathways; thus, future strategies could integrate multi-omics analyses to identify MQC network vulnerabilities with the development of spatiotemporally controllable small molecules (e.g., photocontrolled prodrugs). This approach would shift chemotherapy from an “indiscriminate attack” to a dual-targeted strategy that simultaneously targets tumor cells and protects the MQC system, thereby providing a direct solution to the aforementioned clinical translation barriers (see Table 2).

#### 4.1.1. Tirzepatide

Tirzepatide (TZP), with a molecular formula of C225H348N48O68 and a molecular weight of 4813.45 Da, is a dual-receptor agonist of glucose-dependent insulinotropic polypeptide (GIP) and glucagon-like peptide–1 (GLP–1) [203]. The underlying mechanism is as follows: DOX induces endoplasmic reticulum (ER) stress in cardiomyocytes, upregulates the E3 ubiquitin ligase HRD1, promotes the ubiquitination and degradation of the antioxidant transcription factor Nrf2, and disrupts two key MQC processes—oxidative stress balance and mitophagy [203]. This leads to accumulated damaged mitochondria, enhanced ROS production, and ultimately cardiomyocyte apoptosis. In contrast, TZP inhibits ER stress and HRD1 expression, reduces Nrf2 degradation, enhances its nuclear translocation and transcriptional activity, and upregulates the expression of antioxidant genes such as HO–1. This not only scavenges mitochondrial ROS to restore redox homeostasis (a core MQC component) but also promotes mitophagy of DOX-damaged mitochondria, preventing the collapse of mitochondrial network integrity [187], thereby alleviating myocardial injury. Clinically, DOX is a broad-spectrum antitumor drug, but its clinical application is limited by dose-dependent cardiotoxicity, and there is a lack of effective preventive and therapeutic drugs. In May 2022, the FDA approved tirzepatide for the treatment of type 2 diabetes mellitus (T2DM) [187]. Its definite cardioprotective effect provides a new potential option for the clinical prevention and treatment of chemotherapy-related cardiotoxicity, featuring both safety and application prospects.

#### 4.1.2. Mdivi–1

**Figure 3:**
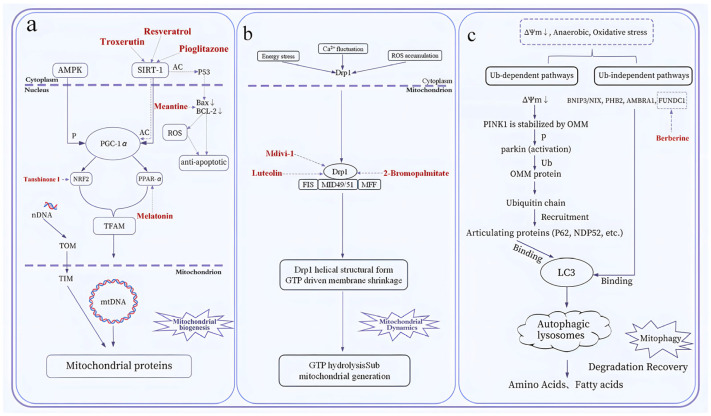
Molecular mechanisms of drug-targeted MQC systems. (**a**) Diagrammatic representation of the classical pathway: drug-induced activation of the AMPK/SIRT1-PGC-1α signaling axis—via AMPK phosphorylation and SIRT1 deacetylation to synergistically activate PGC-1α—promotes interactions between the nuclear genome (NRF2, PPAR-α) and the mitochondrial genome (TFAM), ultimately enhancing mtDNA replication, biosynthesis, and energy production. (**b**) Drug-targeted binding to the GTPase structural domain of Drp1 inhibits its phosphorylation and mitochondrial translocation, preventing mitochondrial membrane contraction and rupture by blocking the assembly of the Drp1-Fis1/Mff/MID49/52 complex. (**c**) Ubiquitin-dependent pathway: bunge seed oil induces loss of ΔΨm↓, promoting accumulation of PINK1 in the mitochondrial outer membrane, phosphorylating ubiquitin, and recruiting Parkin to mediate mitochondrial protein ubiquitination. The ubiquitin chains are recognized by autophagy receptors and bind to LC3-II, forming autophagosomes. Non-ubiquitin-dependent pathway: berberine initiates autophagy by promoting phosphorylation of the FUNDC1 receptor, exposing its LIR domain for direct binding to LC3-II. Both pathways converge into a terminal degradation step: fusion of autophagosomes with lysosomes to eliminate damaged mitochondria. Symbols: →, mechanistic flow; ⇢, drug targeting; ↓, downregulation/inhibition. Solid arrows indicate the directional flow of the core mechanistic pathway; Dashed arrows represent the targeting action of specific drugs.

Mdivi–1 (mitochondrial division inhibitor 1), a cell-permeable selective quinazolinone derivative, specifically mitigates MQC disruption by targeting and repairing the dysregulated mitochondrial dynamics module—a core MQC defect in both DOX-induced cardiotoxicity and oxaliplatin-induced neurotoxicity—through direct inhibition of Drp1, a key GTPase mediating mitochondrial fission [204]. Its primary mode of action involves direct binding to Drp1, inhibiting its enzymatic GTPase activity and preventing the assembly of mitochondrial fission complexes, ultimately reducing oxidative stress and cellular apoptosis [205]. Current studies have shown that Mdivi–1 exhibits therapeutic potential across various pathological conditions, including neuroprotection (e.g., ischemic brain injury, Alzheimer’s disease, Parkinson’s disease), cardiovascular disease (myocardial ischemia/reperfusion injury), antitumor activity (promoting tumor cell apoptosis and enhancing chemotherapy sensitivity), and protection against liver and kidney injury models [206]. Although preclinical studies indicate that Mdivi–1 exhibits a favorable safety profile and effectively penetrates the blood–brain barrier, it has not yet progressed to clinical trials. This underscores the need for further optimization regarding its specificity and dosage control. Future research directions should focus on the development of targeted nanoformulations or prodrugs, the exploration of combination therapies with chemotherapy or immunotherapy, and the expansion of indications to encompass metabolic diseases (Figure 3b).

#### 4.1.3. Pioglitazone

Pioglitazone, a thiazolidinedione-class insulin sensitizer, is primarily used to improve insulin sensitivity in individuals with type 2 diabetes [207]. In recent years, pioglitazone has been found to exhibit agonistic effects on SIRT1, which directly mitigates cisplatin-induced nephrotoxicity by repairing MQC system disruption [182]. Cisplatin directly binds to mitochondrial proteins, impairs mitochondrial respiration, and disrupts two core MQC modules—mitochondrial biogenesis and mitophagy—leading to mtDNA damage, ROS accumulation, and renal proximal tubule cell necrosis [208] (Figure 3a).

#### 4.1.4. Metformin

Metformin, a first-line medication for type 2 diabetes, has emerged as a promising dual-function agent in oncology. Combined with anthracyclines like DOX, it preserves cardiomyocyte mitochondrial function, reduces ROS production, and induces protective autophagy for cardioprotection. Meanwhile, it enhances DOX’s antitumor efficacy via AMPK pathway activation and PD-L1 downregulation in cancer cells. This dual action makes it a valuable candidate for optimizing chemotherapy regimens, balancing therapeutic outcomes and toxicity reduction [176]. Beyond its glucose-lowering effects, recent studies have highlighted its significant “chemo-sensitizing and toxicity-reducing” properties when used in combination with anthracycline chemotherapeutics such as DOX [209]. Metformin exhibits cardioprotective effects by preserving mitochondrial function, reducing the generation of reactive oxygen species (ROS), and inducing protective autophagy in cardiomyocytes. At the same time, it enhances the antitumor efficacy of DOX through the activation of the AMPK pathway and downregulation of PD-L1 in cancer cells. This dual-action characteristic makes metformin a valuable candidate for optimizing chemotherapy regimens, as it aims to maximize therapeutic outcomes while minimizing treatment-related adverse effects [175]. This dual effect (chemo-sensitizing + toxicity-reducing) optimizes the balance between therapeutic efficacy and organ safety.

### 4.2. Natural Products

Natural products offer unique advantages in mitigating chemotherapy-induced toxicities, primarily owing to their multi-targeted modulation of critical MQC pathways (e.g., MB activation, redox homeostasis reconstruction, mitophagy enhancement), with representative compounds and their mechanisms summarized in Table 2. Currently, most remain in preclinical development—only a few derivatives have entered early-phase clinical trials after structural optimization, despite their proven multi-organ protective effects in animal models. While research has elucidated the molecular mechanisms of plant-derived compounds (e.g., resveratrol, troxerutin) in regulating MB via the SIRT1/PGC-1α axis, and these findings have advanced basic research toward clinical translation, such natural products still face inherent limitations for targeting MQC-related multi-organ toxicity: poor bioavailability, rapid in vivo metabolism, unclear overall mechanisms beyond known pathways, lack of organ-specific targeting, and a lack of large-scale clinical trials to validate their safety and efficacy [210] (see Table 2).

#### 4.2.1. Resveratrol

Resveratrol (C14H12O3), a polyphenolic compound derived from plants, exhibits broad chemoprotective effects by targeting the SIRT1–PGC–1α signaling axis to repair MQC disruption [211]. Chemotherapeutics such as DOX and cisplatin suppress SIRT1 activity, leading to impaired mitochondrial biogenesis, enhanced oxidative stress, and collapsed mitochondrial membrane potential. Resveratrol activates SIRT1 to regulate NRF1/TFAM expression for mitochondrial biogenesis and respiratory chain repair [212]. It also enhances SOD and CAT activity via the SIRT1-FOXO3 pathway, reducing mitochondrial superoxide and restoring redox homeostasis [212]. By activating the SIRT1 deacetylase, resveratrol synergistically regulates the expression of genes related to mitochondrial biogenesis and respiratory chain repair, including NRF1 and TFAM. Additionally, the resveratrol-activated SIRT1–FOXO3 pathway enhances the activity of antioxidant enzymes such as superoxide dismutase (SOD) and catalase (CAT), directly reducing mitochondrial superoxide levels and restoring redox homeostasis (a core MQC component) [211].

Experimental evidence suggests that resveratrol prevents the collapse of the mitochondrial membrane potential (ΔΨm), cytochrome c release, and caspase-3 activation, significantly lowering biomarker levels indicative of cellular damage, such as cardiac troponin and serum alanine aminotransferase (ALT). These findings underscore resveratrol’s protective effects against cardiac and renal toxicities by comprehensively repairing MQC system disruption [190] (Figure 3a).

#### 4.2.2. Troxerutin

Troxerutin (C33H42O19), a well-known capillaroprotective agent, effectively mitigates chemotherapy-induced cardiotoxicity, particularly that associated with doxorubicin (DOX), through multifaceted activation of the SIRT1/PGC-1α signaling pathway to repair MQC disruption [213]. Its cardioprotective mechanisms involve enhancing the deacetylase activity of SIRT1, which in turn promotes the PGC-1α-mediated transcription of downstream mitochondrial biogenesis regulators such as NRF1 and TFAM. This process facilitates mitochondrial DNA repair, restores the function of respiratory chain complexes, and reverses DOX-induced mitochondrial network fragmentation, ultimately contributing to its protective effects against cardiotoxicity [64] (Figure 3a).

### 4.3. Nano-Based Drug Delivery System

Traditional chemotherapy remains crucial option for advanced cancer treatment but faces key limitations: poor water solubility of agents (needing toxic co-solvents), lack of tumor specificity (causing off-target toxicities like alopecia), and rapid metabolism with short circulation times [214]. Anti-tumor nano systems (using lipids, polymers, or metals as carriers) address these via small size (20–100 nm), modifiability, and large surface area—improving solubility, extending circulation, and enabling tumor targeting via EPR effect or ligand modification. Currently, most are preclinical, with only a few MQC-targeted formulations (e.g., PMDDH, Ti-mitoEVs) in early trials [215]. Yet they have limitations for MQC-related multi-organ toxicity: insufficient organ-specific targeting, potential biocompatibility risks (e.g., inflammation), inconsistent large-scale production, and unclear impacts on chemotherapy’s antitumor efficacy(see Table 2).

#### 4.3.1. PMDDH

PMDDH, a novel self-assembled nanomedicine, is specifically designed for the co-delivery of DOX and metformin to synergistically enhance antitumor efficacy and repair MQC disruption-mediated cardiotoxicity [176]. This innovative formulation not only enhances the pharmacokinetics and tumor-targeting capabilities of DOX but also preserves the biological activity of metformin, leading to a synergistic increase in anti-tumor efficacy while reducing cardiotoxicity (see Table 2). Notably, PMDDH further mitigates DOX-induced cardiotoxicity by diminishing reactive oxygen species (ROS) production, preserving mitochondrial function, and inducing protective autophagy through the AMPK–mTOR signaling pathway. As a result, it achieves significant cardioprotective effects in vivo [176].

#### 4.3.2. Ti–MitoEVs

Ti–mitoEVs, tissue-derived mitochondria-rich extracellular vesicles isolated from healthy skeletal muscles, offer an innovative nanotherapeutic approach for MQC repair in damaged tissues [9]. These vesicles are enriched with intact mitochondrial genomes, ETC proteins, and MQC regulatory factors—addressing the root cause of chemotherapy-induced toxicity: severe MQC disruption (e.g., mtDNA depletion, ETC dysfunction, impaired biogenesis) [9]. Their core mechanism lies in the direct delivery of functional mitochondrial components to recipient cells via mitochondrial genome transfer: on one hand, they increase mtDNA copy number and the expression of mitochondria-encoded genes (e.g., ND1, Cytb) to promote mitochondrial biogenesis and restore OXPHOS function; on the other hand, they inhibit oxidative stress (reducing ROS production) and damage-related inflammation (decreasing macrophage infiltration and cytokine release), thus reversing MQC collapse-mediated cell death and blocking the process of tissue fibrosis [9]. In vitro and in vivo experiments have confirmed that Ti–mitoEVs can significantly alleviate acute muscle injury (attenuating myofiber lysis and restoring mitochondrial networks) and chronic kidney disease (reducing renal fibrosis and improving mitochondrial quality) in models of cisplatin- or doxorubicin-induced toxicity. Moreover, their natural phospholipid bilayer structure protects encapsulated cargos from environmental damage, endowing them with superior biosafety and targeting capabilities compared to traditional mitochondrial transplantation or small-molecule drugs. This therapeutic strategy provides a new option for mitochondrial damage-related diseases in multiple organs such as skeletal muscle and kidneys, holding broad potential for clinical translation [9].

## 5. Conclusions

This review establishes that chemotherapy-induced multi-organ toxicity arises from selective disruption of the mitochondrial quality control (MQC) network. We propose an integrated framework encompassing all five core MQC components—highlighting the recently identified migrasome-mediated mitocytosis pathway—to explain the convergent injury mechanisms across organs. Chemotherapeutics coordinately impair MQC by suppressing the PGC-1α/SIRT1/SIRT3 axis, hyperactivating Drp1 with MFN2 downregulation, blocking PINK1/Parkin-mediated mitophagy, disrupting HSP60/LONP1 proteostasis, and inhibiting migrasome-mediated mitocytosis. These disruptions collectively cause mitochondrial ROS overflow, energy failure, and cell death. Delineating organ-specific MQC pathways—such as cardiac TOP2β, neuronal CaMKII, and renal DHODH-associated ferroptosis—provides a molecular basis for precision organ protection.

By establishing a unified paradigm of “MQC dysfunction” and outlining actionable therapeutic roadmaps, we seek to bridge mechanistic insights with clinical translation [158]. The core challenge, however, remains balancing chemotherapy’s antitumor efficacy against its off-target toxicity—a difficulty rooted in the divergent MQC adaptability between tumor cells and normal tissues. While tumors rely heavily on functional MQC for proliferation, normal cells possess compensatory mechanisms like migrasome-mediated mitocytosis and UPRmt activation [216]. However, chemotherapy-induced multi-target MQC disruption overwhelms these defenses, leading to organ injury. Thus, achieving “bifunctional modulation”—intensifying MQC disruption in tumors while reinforcing MQC homeostasis in normal tissues—could redefine chemotherapy’s therapeutic window [158]. Key strategies for implementing this paradigm include (1) elucidating the fundamental distinctions between the mitochondrial homeostasis of specific tumor cells and that of chemotherapy-vulnerable organs [217]; (2) temporal stratification of mitochondrial clearance complex regulation, such as preserving mitochondrial reserve capacity during neoadjuvant therapy and repairing mitophagy during adjuvant treatment; (3) dose titration based on real-time mitochondrial clearance complex responsiveness; and (4) optimization of precision-targeted delivery systems, including organ-specific nanocarriers and tissue-directed molecular switches. Furthermore, leveraging compensatory crosstalk between MQC modules—such as the role of migrasome-mediated mitocytosis as a backup clearance pathway in normal cells when mitophagy is impaired—offers a novel avenue to decouple antitumor efficacy from off-target toxicity by exploiting the functional redundancy of MQC in healthy tissues, a feature largely absent in tumors.

Given the distinct growth and developmental characteristics of children—and the substantial differences between pediatric and adult tumors—research on the MQC system in relation to treatment-related toxicities in pediatric oncology remains notably limited. This knowledge gap significantly constrains the translation of MQC-targeted strategies to mitigate side effects in childhood cancer patients.

## Figures and Tables

**Table 1:** Brief comparison of organ-specific MQC dysregulation induced by chemotherapy.

Organ	Chemotherapeutic Agent	Core MQC Impairment	Organ-Specific Molecules	Typical Pathological Feature
Heart	Doxorubicin	1. DOX upregulates TLR5 → Syk/PLCγ1/PKCαactivation → p47phox translocation → NOX2 complex assembly → superoxide excess; cardiomyocytes have low antioxidant capacity (reduced GCLC) [53]; 2. DOX binds TOP2β to form complex → blocks PGC-1α/β promoters; p53 activation represses PGC-1α/β → inhibits NRF1/TFAM-mediated MB [168]; 3. Reduced Mfn1/2/OPA1 (impaired fusion); ROS/PKCδ-mediated Drp1-Ser616 phosphorylation [74]; 4. p53 sequesters Parkin → blocks PINK1/Parkin pathway; hnRNPK downregulation represses PINK1 transcription [56]; 5. HSP60/HSP10 dysfunction → protein misfolding; LonP1 downregulation → abnormal protein accumulation; YME1L1/OMA1 imbalance → OPA1 cleavage disorder [169].	PGC-1α, Drp1, Mfn2, TLR5-NOX2, HSP60/LONP1.	Left ventricular dysfunction; dilated cardiomyopathy; heart failure; cardiomyocyte apoptosis/necrosis; delayed cardiac injury (more prevalent in pediatric patients)
Neurotoxicity	Oxaliplatin	1. Oxidative stress: Oxaliplatin (OCT2-mediated DRG accumulation) inhibits mitochondrial ETC (I/III) → superoxide excess; oxalate enhances TRPM8 currents [170,171]; 2. Mitochondrial dynamics: Reduced MFN2 (impaired fusion); CaMKII-mediated Drp1-Ser616 phosphorylation (excessive fission) [172,173]; 3. Blocked mitophagic flux: Transient PINK1/Parkin activation → Parkin suppression; autophagosome-lysosome fusion stall [129];	TRPM8, MFN2, CaMKII, Parkin, PGC-1α.	Length-dependent “glove-and-stocking” pattern of sensory abnormalities; cold hypersensitivity; acute reversible/chronic dose-dependent neurotoxicity; axonal transport failure
Kidney	Cisplatin	1. Cisplatin (OCT2-mediated PTC accumulation) induces ROS → Drp1 translocation/activation; reduces MFN1/2 → fusion impairment; fragmented mitochondria release cytochrome C [157]; 2. Transient PINK1/Parkin activation → subsequent autophagosome-lysosome fusion stall; damaged mitochondria accumulate (TOM20 ↑), triggering inflammation/apoptosis [174]; 3. Cisplatin represses PGC-1α-NRF1/TFAM → mitochondrial regenerative capacity loss; compromises PTC energy repair [17]; 4. ROS induces DHODH acetylation (SIRT3 ↓) → CoQH2 depletion, upregulates PUFA-PLs (ACSL4/LPCAT3), and inhibits GPX4/FSP1 → lipid peroxidation accumulation [146].	Drp1, MFN2, DHODH, GPX4, PINK1/Parkin, PGC-1α, NAD^+^ precursors.	Acute tubular necrosis; decreased glomerular filtration rate; edema/necrosis of renal tubular epithelial cells; electrolyte imbalance; dose-dependent nephrotoxicity

Note: ↑, upregulation or increase; ↓, downregulation or decrease.

**Table 2:** Pharmacological modulation of MQC systems to protect against chemotherapy-induced injury.

Intervention Type	Representative Drugs/ Compounds	Target	Core Mechanisms	General Toxicity Type	Clinical Stage	References
Small-molecule compounds	Metformin	Drp1, OPA–1	It downregulates Drp1 and upregulates OPA–1, while also conferring antioxidant and anti-apoptotic effects.	Doxorubicin-induced cardiotoxicity	Phase II (failed); preclinical (investigational)	[175]
	PMDDH	AMPK/mTOR	Co-delivery of DOX and metformin.	Doxorubicin-induced cardiotoxicity	Preclinical (novel nanomedicine)	[176]
	MODICA	VDAC	Inhibition of VDAC polymerization	Doxorubicin-induced cardiotoxicity	Preclinical	[177]
	Dexrazoxane	Top2β/Iron Chelation	Blocks DOX–Top2β binding and chelates free iron to mitigate Fenton reactions, preserving mtDNA integrity.	Anthracycline cardiotoxicity	Approved	[178]
	Mdivi–1	Drp1	Inhibits phosphorylation of Drp1 to suppress excessive mitochondrial fragmentation and restore dynamics (Figure 3b).	Neurotoxicity	Preclinical	[179]
	Amifostine	ROS clearance/Platinum chelation	Eliminates ROS selectively in normal tissues, reducing mitochondrial accumulation of cisplatin.	Cisplatin nephrotoxicity	Approved for clinical use	[180]
	PDE10A inhibitors	PDE10A/cAMP–cGMP	Elevates cAMP–cGMP levels, activating PKA to enhance antioxidant enzyme activities (SOD/GPx) and mitigate mitochondrial ROS bursts.	Anthracycline cardiotoxicity	Preclinical	[181]
	Pioglitazone	SIRT1/p53	Alleviates cisplatin nephrotoxicity by regulating SIRT1/p53-mediated mitochondrial apoptosis (Figure 3a).	Cisplatin nephrotoxicity	Preclinical	[182]
	Memantine	Bax/Bcl–2, Caspase–3	Reduces mitochondrial-dependent neurotoxicity induced by oxaliplatin (Figure 3a).	Oxaliplatin neurotoxicity	Preclinical	[183]
	2–Bromopalmitate	Drp1	Reduces Drp1-mediated mitochondrial dysfunction, attenuating oxaliplatin-induced neuropathic pain (Figure 3b).	Oxaliplatin neurotoxicity	Preclinical	[124]
	PEA	NF–κB/Nrf–2	Alleviates oxaliplatin-induced painful neuropathy via modulation of NF–κB/Nrf–2 signaling.	Oxaliplatin neurotoxicity	Preclinical	[184]
	Lasmiditan	5–HT1F receptor	Indirectly modulates mitochondrial stability through regulation of PGC–1α, AMPK, and fusion/fission proteins, protecting against renal ischemic injury.	Kidney injury	Preclinical	[185]
	Salvianolic Acid B	AMPK/SIRT1/PGC–1a	Reduces kidney injury by activating the AMPK/SIRT1/PGC–1α signaling axis.	Kidney injury	Preclinical	[186]
	Tirzepatide	HRD1, Nrf2	Mitigates DOX cardiotoxicity by inhibiting ER stress/HRD1, stabilizing Nrf2, and enhancing HO–1 expression.	Doxorubicin-induced cardiotoxicity	Preclinical	[187]
Natural Products	Melatonin	PPARα	Prevents acute kidney injury induced by cisplatin via upregulation of PPARα expression (Figure 3a).	Cisplatin nephrotoxicity	Phase II clinical trial	[188]
	Berberine	FUNDC1	Protects mitochondrial networks in glomerular podocytes by downregulating FUNDC1 expression (Figure 3c).	Cisplatin nephrotoxicity	Preclinical	[189]
	Resveratrol	SIRT1/PGC–1α	Activates SIRT1–PGC–1α pathway, promoting MB and enhancing antioxidant defenses (Figure 3a).	Multi-organ protection (cardiac/renal/neuro)	Preclinical	[190]
	Honokiol	SIRT3 activator	Prevents ROS production, mitochondrial damage, and cell death induced by DOX in neonatal rat cardiomyocytes through activation of SIRT3.	Anthracycline cardiotoxicity	Preclinical	[191]
	Mangiferin	Nrf2/HO–1	Enhances antioxidant Nrf2/HO–1 signaling to maintain redox balance.	Reduces kidney ischemia/reperfusion injury	Preclinical	[192]
	Ginsenoside F1	Nrf2/HO–1	Inhibits ferroptosis via increased HO–1 expression, promoting free iron clearance.	Anthracycline cardiotoxicity	Preclinical research stage	[193]
	Tanshinone I	Nrf2	Attenuates oxidative stress by regulating Nrf2 signaling (Figure 3a).	DOX cardiotoxicity	Preclinical	[194]
	Glycyrrhetinic Acid	Nrf2/HO–1	Suppresses oxidative stress, mitochondrial dysfunction, and apoptosis via the Nrf2/HO–1 pathway.	DOX cardiotoxicity	Preclinical	[195]
	Silibinin	SIRT3	Enhances mitochondrial function by modulating SIRT3 expression.	Cisplatin nephrotoxicity	Preclinical research (animal/cellular studies)	[196]
	Tanshinone IIA	PI3K/Akt/mTOR	Promotes autophagy via activation of the PI3K/Akt/mTOR pathway.	Oxaliplatin peripheral neurotoxicity	Preclinical	[197]
	Luteolin	Drp1	Suppresses mitochondrial fragmentation and oxidative stress by inhibiting Drp1 phosphorylation.	Anthracycline cardiotoxicity	Preclinical	[56]
	Rosmarinic Acid	AMPK	Reduces oxidative stress and enhances cellular energy metabolism via AMPK activation in peripheral nerves and dorsal root ganglia (Figure 3a).	Platinum-induced peripheral neuropathy	Preclinical	[198]
	WGX50	ROS, Ferroptosis	Attenuates DOX-induced cardiac damage through inhibition of mitochondrial ROS and ferroptosis.	DOX cardiotoxicity	Preclinical candidate	[199]
	Troxerutin	SIRT1/PGC–1a	Enhances MB by activating the SIRT1/PGC–1α pathway (Figure 3a).	Alleviation of DOX-induced myocardial injury and oxidative stress.	Preclinical	[64]
	Gastrodin	ROS, SIRT1	Mitigates cisplatin nephrotoxicity by reducing ROS generation and increasing SIRT1 expression.	Cisplatin nephrotoxicity	Preclinical	[200]
	Pterostilbene	PGC–1α	Reduces oxidative stress by activating PGC–1α, enhancing AMPK and SIRT1 signaling cascades.	Cardiac injury	Preclinical candidate	[201]

## Data Availability

No data was used for the research described in the article.
